# Effect of Cigarette Smoke on Wound Healing of the Septal Mucosa of the Rat

**DOI:** 10.1155/2016/6958597

**Published:** 2016-03-02

**Authors:** Veronica Trombitas, Alina Nagy, Cristian Berce, Flaviu Tabaran, Silviu Albu

**Affiliations:** ^1^2nd Department of Otolaryngology, Iuliu Hatieganu University of Medicine and Pharmacy, 18 Republicii, 400015 Cluj-Napoca, Romania; ^2^Department of Experimental Medicine, Iuliu Hatieganu University of Medicine and Pharmacy, 18 Republicii, 400015 Cluj-Napoca, Romania; ^3^Pathology Department, University of Agricultural Sciences and Veterinary Medicine, Cluj-Napoca, Romania

## Abstract

*Objectives/Hypothesis*. Proper wound healing following endoscopic sinus surgery (ESS) is influenced by several factors, like cigarette smoke (CS) exposure. This study aims to assess the influence of cigarette smoke on the healing of induced septal mucosal lesion in rats.* Methods*. Unilateral nasal wounds were created by means of the interdental brush in seventy-four-week-old male rats. Animals were randomly divided into two groups: control group and CS exposure group, each comprising 35 animals, divided into five groups (*n* = 7). Animals were sacrificed in groups of seven on day 2 and then on days 5, 14, and 28 and finally on day 42 following wound induction.* Results*. Histological analysis of mucosal specimens shows important changes at the CS exposure group. Starting with the infiltrates of neutrophils, eosinophils, macrophages, and lymphocytes, the histological changes were continued with the Goblet cell proliferation, ciliated cells loss, fibrosis, and epithelial and subepithelial hypertrophy.* Conclusion*. In this experimental model of nasal wound healing we demonstrated the deleterious effects of chronic CS exposure. The adverse effects of CS exposure are firstly a postponement of the healing process and secondly the persistence of inflammation which becomes chronic.

## 1. Introduction

Because of the increasing prevalence in the general population, chronic rhinosinusitis (CRS) has an important economic impact. In addition genetic factors, allergy, inflammation, and environmental factors are among the risk factors associated with CRS pathogenesis. Besides environmental pollution, other risk factors associated with CRS are active smoking and secondhand smoke (SHS) [1]. SHS is defined as a combination of breathing out mainstream smoke arising from the smoker and side stream smoke from the burning end of a cigarette. It is estimated that 35% female nonsmokers, 33% male smokers, and 60% children aged 3–11 years are commonly exposed to SHS. There is a strong association between smoking and the increased incidence of upper airway inflammatory disease in both adults and children [1–3]. Moreover, active smoking and SHS are predictive factors associated with recurrent disease following ESS in CRS [4–7].

ESS remains the gold standard for the treatment of CRS, especially for the recalcitrant CRS associated with active smoking, asthma, and allergies [8, 9]. Wound healing after ESS represents an important process that includes inflammation, formation of granulation tissue, and finally remodeling of new tissue to restore tissue integrity [9]. All these stages are regulated by cytokines and growth factors and also could be disturbed by factors that induce local hypoxia or low-nutrition [9, 10].

Because of the inherent limitations in studying wound healing following ESS in humans, animal models have emerged as a powerful tool to apprehend wound repair pathogenesis [9]. The rat model has been preferred in the study of nasal mucosa wound healing for several benefits: analogous respiratory epithelium to humans, small size, and accelerated wound healing [11]. Thus, this model was acknowledged to describe the evolution of postoperative wound healing following ESS [11]. It is acknowledged that rats exposed for a long time to cigarette smoke (CS) develop upper airway histological changes (like emphysema, also goblet cell metaplasia especially in small bronchioles, and sometimes vascular remodeling) and functional abnormalities [10, 11].

This study aims to assess the influence of cigarette smoke on the healing of induced septal mucosal lesion in rats.

## 2. Materials and Methods

### 2.1. Animal Preparation

For the purpose of the study we used seventy-four-week-old male rats with no evidence of respiratory disease. All the experiments conformed to the Guidelines of the National Institutes of Health and the Declaration of Helsinki and were approved by the Committee on the Use and Care of Animals at the Iuliu Hatieganu University of Medicine and Pharmacy, Cluj-Napoca. Animals were examined by a veterinarian and the diet and water were given ad libitum, temperature was 21 degrees, and animals had day-night cycle of 12/12 hours.

A mechanical injury was induced as described: following anesthesia with intraperitoneal injection of Zoletil 50 (10 mg/kg), a unilateral nasal wound was produced by means of the interdental brush introduced through the right nostril. The animals were randomly divided into two groups: control group and CS exposure group, each comprising 35 animals, divided into five groups (*n* = 7).

Seven rats from each group were killed on days 2, 5, 14, 28, and 42 subsequent to the injury and their heads were removed. Each head was placed in a separate envelope marked with a certain code known by only the principal investigator. The code was hidden until histologic examinations of the specimens were completed. The pathologist performing histological analysis was blinded to the study group. The rats' heads were prepared through elimination of skin, removing of eyes, muscle, and the mandibles. The heads were submerged in Bouin's fluid for one night and further they were retained in 5% nitric acid for 5–7 days. As suggested, the samples were collected from the posterior area of the upper incisors at the level of incisive papilla. Tissues were initially dehydrated and then were processed through the paraffin-embedding techniques. Histological analysis was realized using H&E and Masson's trichrome staining. In the control group, the animals have nasal wounds, but they are not exposed to CS.

### 2.2. CS Exposure

The protocol described by Ardite et al. [12] was precisely followed in our study: the rats were placed within the custom-made smoking chamber on an acryl plate. The cigarette was positioned at the end of a conduit attached to the animal ventilator. CS exposure was 1 h/day and 5 days/week in the afternoon. CS exposure animals were divided into five groups (*n* = 7); each group was exposed to both mainstream and side stream smoke. Mainstream smoke consisted in smoke dragged with the help of ventilator and redirected into the chamber, while smoke released directly from the burning end of the cigarette was considered side stream smoke. One exposure was represented by the exposure to one commercial cigarette every 12 minutes for 1 hour (totally 5 cigarettes) [13, 14].

### 2.3. Histological Analysis

Because the normal wound healing includes inflammatory reaction, immune response, tissue remodeling, and maturation [9], histological examination was centered on inflammatory cell infiltrates, edema, goblet and ciliated cells, subepithelial fibrosis, and epithelial and subepithelial thickness. All these histological changes were observed and compared on days 2, 5, 14, 28, and 42 after mechanical injury between CS exposed group and control group.

In conformity with Khalmuratova study [9, 11] evaluation of goblet and ciliated cells was made through counting, calculating the ratio of newly formed cells (for goblet cells), or just the number of cells (for ciliated cells) in the H&E-stained section at 400x magnification between the injured and contralateral nasal mucosa for CS exposed and control groups. On the other hand Epithelial Thickness Index (ETI) represents the ratio between the height of the regenerated epithelium (for the injury site) and the height of the contralateral epithelium [9, 11] (average of three measures) for both experimental groups, in H&E-stained sections at 200x magnification. The Subepithelial Thickness Index (STI) was calculated following the same technique as for ETI, in H&E-stained sections at 200x magnification. An STI value of ≥1.0 was considered hypertrophic subepithelium [9, 11].

The subepithelial fibrosis index (STI) was used to measure the pathological fibrosis identified on H&E and Masson's trichrome, stained sections at 200x magnification. Eosinophils were counted on Luna's stain, periodic acid–Schiff (PAS) stain was employed for goblet cells, and toluidine-blue stain was used for determining mast cells. Measurements were taken from five subepithelial areas on the experimental and control specimens. It was considered that SFI ≤ 1.0 is equivalent to control and >1.0 represents fibrosis [9, 11].

Morphometric evaluations were realized with an image analysis device (Olympus BX51, “Olympus Soft Imaging solution cell B”) and images were captured using the Olympus DP-25 digital camera.

### 2.4. Statistical Analysis

Results were analyzed using SPSS software (SPSS, Inc., Chicago, IL). The differences between CS and control groups were evaluated using the nonparametric Mann-Whitney and MANOVA tests. The figures have been presented as the mean associated or not with standard deviation. A *p* < 0.05 value was considered significant.

## 3. Results

**Figure 1:**
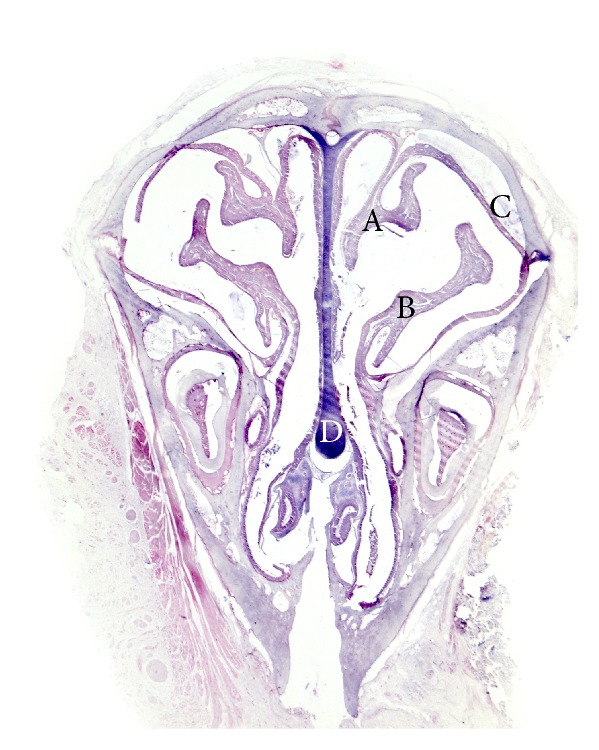
Sinosal cavity in coronal section of rat model. (A) Nasoturbinate, (B) maxilloturbinate, (C) lateral nasal wall, and (D) nasal septum (Masson's trichrome stain).

Mucosal specimens were obtained from the nasal septum in the middle third of the sinonasal cavity using coronal sections (Figure 1). Microscopic analysis of mucosa specimens shows significant histological alterations within the CS exposure group, both in the epithelium and in the connective tissue, respectively, in the glandular tissue associated with lamina propria. In the early stages CS generates injury solely in the surface epithelium, while later lesions expand also to the lamina propria. The surface epithelium displays progressive alteration consisting initially in cilia loss and goblet cell hyperplasia, followed by hyperplasia or dysplasia.

### 3.1. Histological Evolution Related to Time

**Figure 2:**
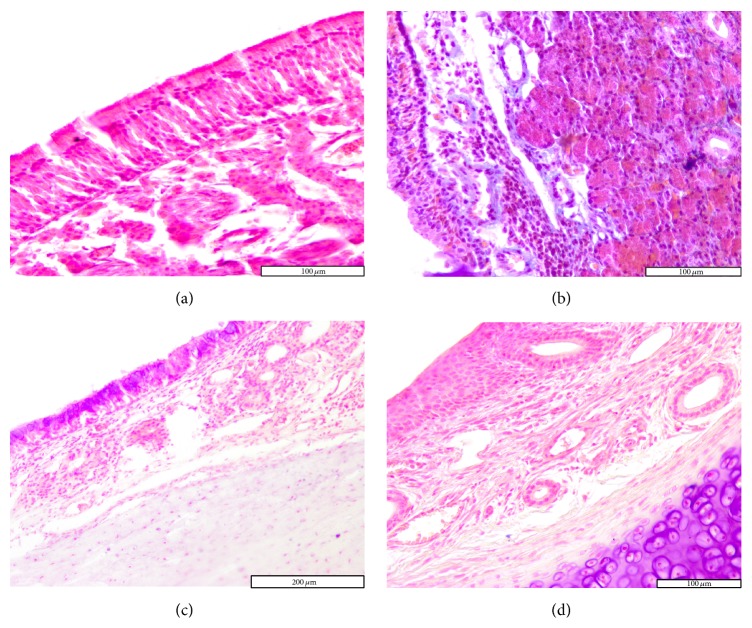
Photomicrographs of the rat septal mucosa, 2 and 5 days after injury, with or without cigarette smoke exposure. (a) Minimal infiltrate with neutrophils and lymphocytes, after 2 days of cigarette smoke exposure. (b) Multifocal dysplasia after 2 days in the control group. (c) Goblet cell hyperplasia and epithelial dysplasia, after 5 days of cigarette smoke exposure. (d) Hypertrophy of the mucosal glands in the experimental group after 5 days (hematoxylin and eosin stain; original magnification ×100, ×200).

On day 2 congestion and hemorrhage in the nasal mucosa were noted in the exposed group. Histology highlights the presence of multifocal infiltrates of neutrophils, eosinophils, macrophages, and lymphocytes. Occasionally necrotic lesions were witnessed, represented by desquamated cells with nuclei in the pyknotic or karyorrhexis stages. This feature is coined as “single cell necrosis.” On septum epithelium focal hyperplasia and areas with absent cilia were predominant (Figure 2(a)).

**Figure 3:**
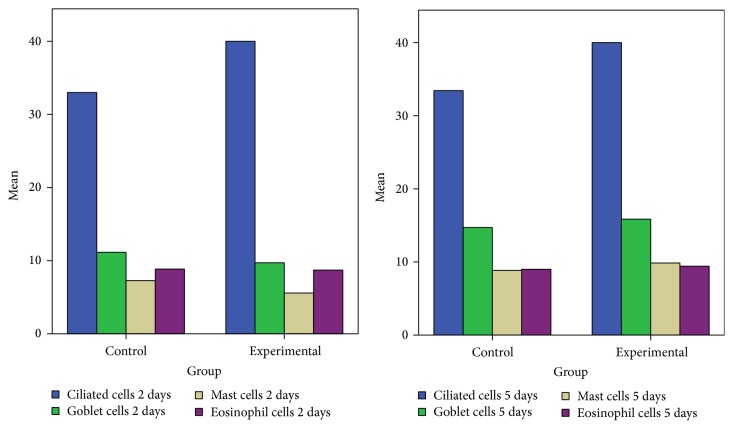
Eosinophils, goblet and mast cells distribution in the two groups (days 2 and 5 after injury).

The differences with the control group were minimal, since in the control group the congestion, hemorrhage, and multifocal necrosis were predominant. Microscopic examination displayed neutrophils, eosinophils, macrophages, and lymphocytes infiltrate (Figure 2(b)). The numbers of eosinophils (Figure 10), mast cells (Figure 8), and goblet cells (Figure 9) were not different between the two groups as demonstrated in Figure 3.

Goblet cell proliferation in nasal epithelium and the presence of fibrous connective tissue with a rich cellular network were observed on day 5 in the experimental group. Nasal mucosa associated glands were hypertrophied and had a large lumen (Figures 2(c) and 2(d)). Intraepithelial dysplasia with cilia disappearance has also been observed. Leukocyte infiltration was reduced quantitatively. The control group showed ortokeratotic keratinization on the nasal epithelium. Lamina propria presented fibroplasia, categorized by a young connective tissue with many cells. However, inflammatory cell counts were not different between the groups (Figure 3).

**Figure 4:**
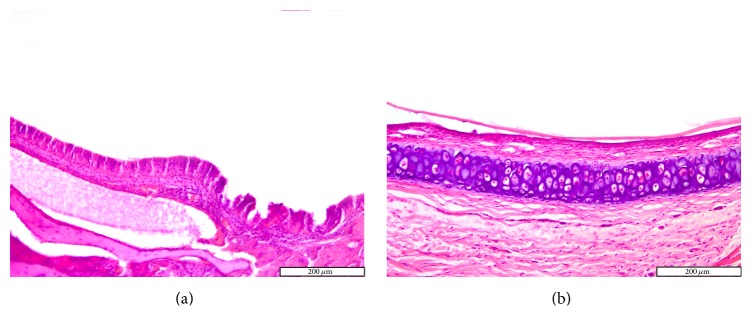
Photomicrographs of the rat septal mucosa after 14 days of injury, with or without cigarette smoke exposure. (a) Epithelial thickness and micropapillary structures after 14 days of cigarette smoke exposure. (b) Control group displays squamous metaplasia (hematoxylin and eosin stain; original magnification ×100, ×200).

**Figure 5:**
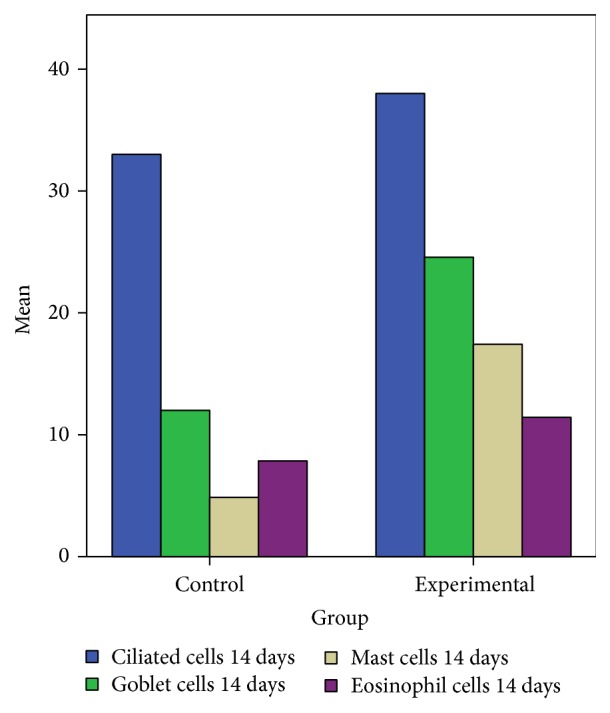
Eosinophils, goblet and mast cells distribution in the two groups 14 days after injury. Significant increase of eosinophils, goblet and mast cells after injury and cigarette smoke exposure.

On day 14 significant goblet gland hyperplasia was witnessed in the experimental group (*p* < 0.05; see Figure 5) and moderate fibroplasia in the lamina propria. Occasionally the lamina propria displayed a nodular infiltrate consisting in polymorphonuclear (mainly eosinophils and rare neutrophils) and lymphocytes. Polypoid protrusions supported by a core of connective tissue were noted on the nasal epithelium (Figure 4(a)). The ETI and STI differences between the two groups were statistically significant (*p* < 0.05, MANOVA test) (Figures 13 and 14). The control group presented squamous metaplasia and keratinization. In the lamina propria was observed moderate fibrosis (Figure 4(b)). The number of eosinophils (Figure 10), mast cells (Figure 8), and goblet cells (Figure 9) was significantly increased in the CS exposed group (see Figures 5 and 12).

**Figure 6:**
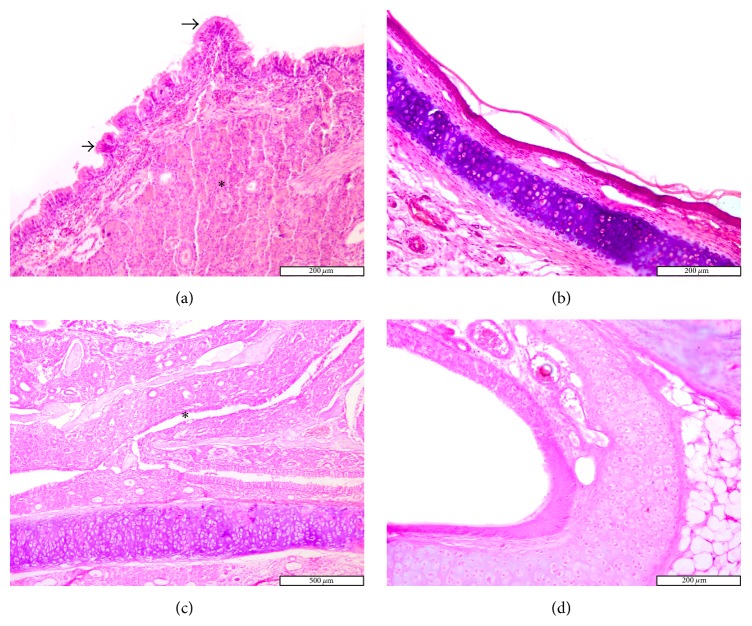
Photomicrographs of the rat septal mucosa, 28 and 42 days after injury, with or without cigarette smoke exposure. (a) Experimental group: nasal mucosa hyperplasia, possible prepolypoid changes (long and short arrow), and goblet cell proliferation (*∗*) after 28 days of cigarette smoke exposure. (b) Control group: minimal nasal epithelium squamous metaplasia, 28 days after injury. (c) Epithelial thickness with goblet cell proliferation and important obstruction (*∗*), after 42 days of cigarette smoke exposure. (d) Squamous metaplasia in the control group, 42 days after injury (hematoxylin and eosin stain; original magnification ×100, ×200).

**Figure 7:**
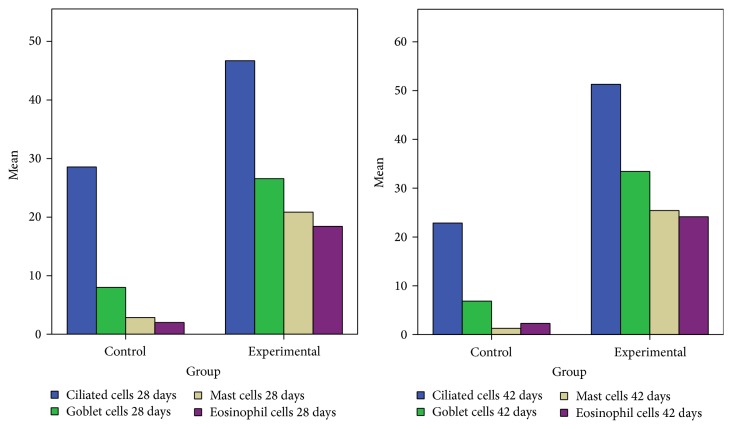
Eosinophils, goblet and mast cells distribution in the two groups 28 and 42 days after injury. Significant increase of eosinophils, goblet and mast cells after injury and cigarette smoke exposure.

**Figure 8:**
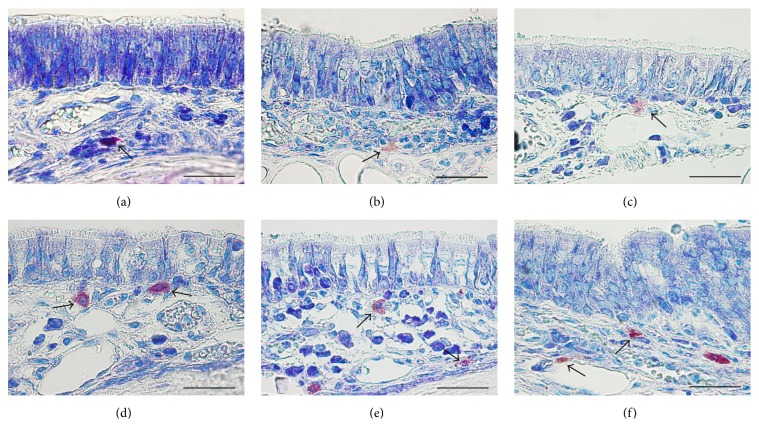
Micrographs showing toluidine-blue-stained sections of nasal septum mucosa; (a) represents the control group, while (b), (c), (d), (e), and (f) present the mast cell population at 2, 5, 14, 28, and, respectively, 48 days subsequent to injury. Metachromatic-stained mast cells from septal mucosa lamina propria are indicated by the arrows; toluidine blue, Obx100; scale bar = 50 *μ*m.

**Figure 9:**
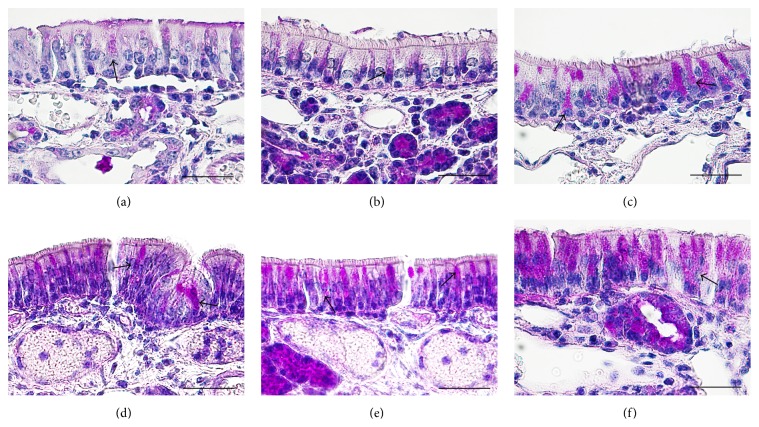
Micrographs showing periodic acid–Schiff-stained sections of nasal cavity; (a) represents the control group, while (b), (c), (d), (e), and (f) present the PAS-stained mucosubstances at 2, 5, 14, 28, and, respectively, 48 days subsequent to septal injury. PAS positive goblet cells from nasal mucosa are indicated by the arrows; periodic acid–Schiff, Obx100; scale bar = 50 *μ*m periodic acid-Schiff stain.

**Figure 10:**
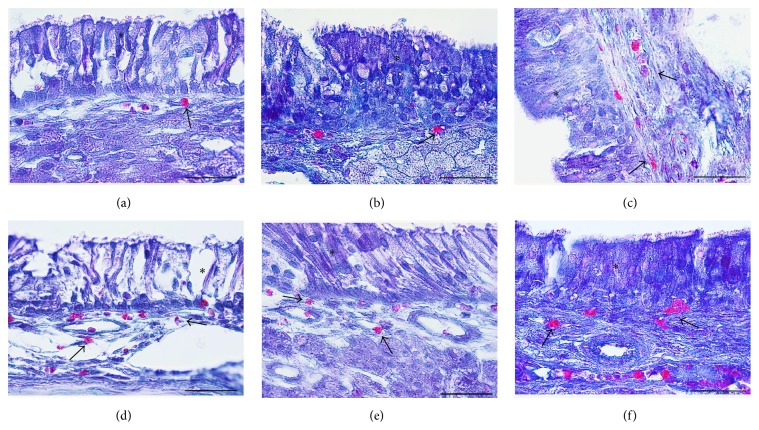
Histopathological images showing infiltrative eosinophils scattered in nasal mucosa; (a) represents the control group, while (b), (c), (d), (e), and (f) present the eosinophils infiltration at 2, 5, 14, 28, and, respectively, 48 days subsequent to injury. Eosinophils with characteristic orange-red granular content are indicated by the arrows; the asterisks indicate the nasal respiratory mucosa; Luna's stain for eosinophils, Obx100; scale bar = 50 *μ*m.

On the 28th day in the CS group the alterations were already chronic, both in the epithelium and in the lamina propria. The surface nasal epithelium showed multifocal hyperplasia and papillary proliferations with a supportive connective tissue stroma. The histological changes in the lamina propria consisted of marked hypertrophy of glands, fibroplasia for the connective tissue, and infiltrates with eosinophils, lymphocytes, respectively, rare neutrophils. The histological features presented designate a chronic inflammation of the nasal mucosa (Figure 6(a)). The number of eosinophils (Figure 10), mast cells (Figure 8), and goblet cells (Figure 9) was significantly increased in the experimental group (see Figures 7 and 12).

**Figure 11:**
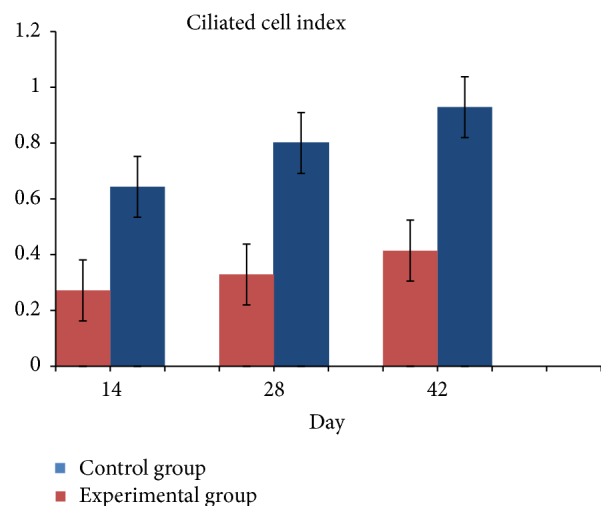
Ciliated cell index. The values were expressed as the mean ± SEM (*p* < 0.05).

**Figure 12:**
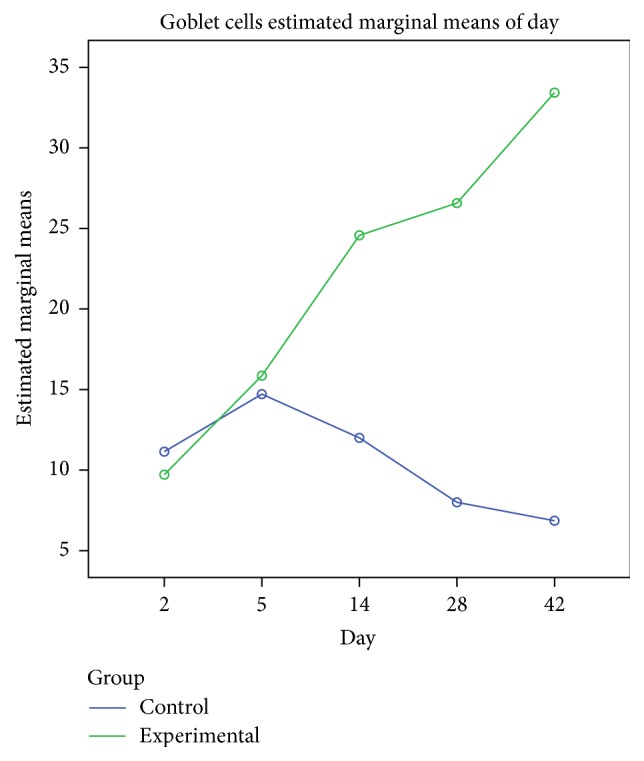
Goblet cell index over time, comparison with MANOVA test between the two groups. Significant difference at days 14, 28, and 42.

**Figure 13:**
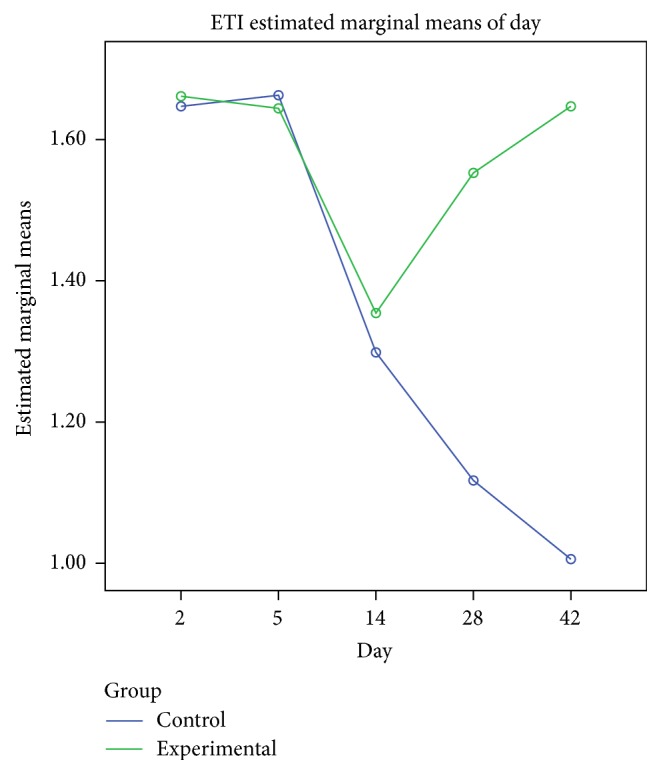
Epithelial fibrosis index (EFI) comparison with MANOVA between the two groups. Significant difference at days 28 and 42 following injury.

**Figure 14:**
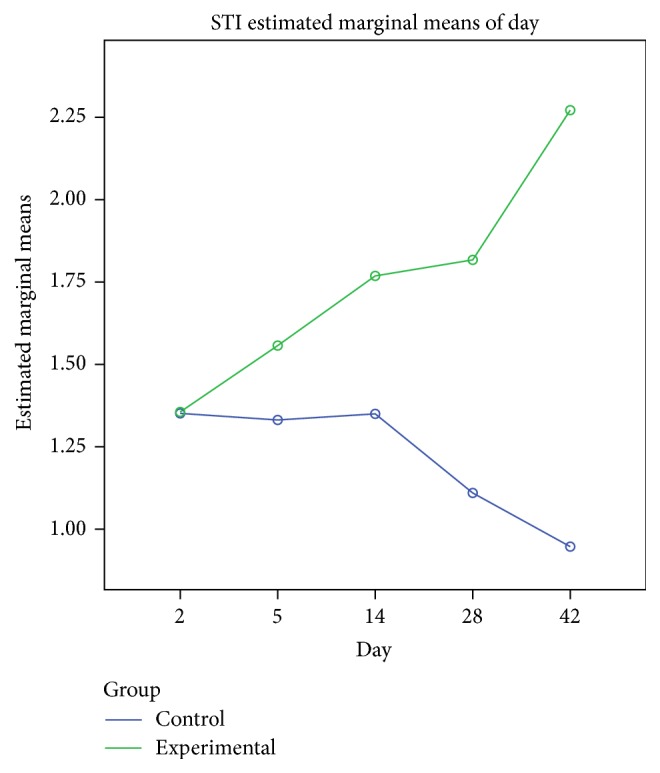
Subepithelial fibrosis index (SFI), comparison between the two groups with MANOVA test. Significant difference at 5, 14, 28, and 42 days.

By day 42 the chronic alterations were represented by marked hyperplasia in the lamina propria (Figure 6(c)), epithelial hyperplasia and dysplasia, and goblet cell hyperplasia (*p* < 0.05). The loss of ciliated cells was significant in the injury site (*p* < 0.05), (Figure 11). The number of eosinophils (Figure 10), mast cells (Figure 8), and goblet cells (Figure 9) was significantly increased in the experimental group (Figures 7 and 12). In certain areas, associated glands were much hypertrophied and they had a wide lumen. Leukocyte infiltrate was discrete and an eosinophilic infiltrate was also present. The number of eosinophils (Figure 10), mast cells (Figure 8), and goblet cells (Figure 9) was significantly increased in the experimental group (Figures 7 and 12).

On days 28 and 42, the control group displayed hypertrophy and a background of fibroplasia in the lamina propria. Epithelial alterations were represented by dysplasia, rarely cilia loss, and goblet cell proliferation. Occasionally even squamous metaplasia was encountered, including surface keratinization (Figures 6(b) and 6(d)). All histological changes were minimal and represent a normal nasal mucosa healing pattern.

## 4. Discussion

Proper wound healing following ESS is influenced by several internal and external factors [15]. This compound process comprises inflammation, granulation tissue development, and tissue remodeling embracing cell proliferation, angiogenesis, and reepithelization [16]. Any interference with the natural healing process may induce morphological and physiological alterations of the sinus mucosa [15, 16].

It has been demonstrated that recurrent rhinosinusitis is more prevalent among smokers compared to nonsmokers [4–7]. Several studies have certified the deleterious influence of CS on ESS outcomes: active smokers have poor symptomatic, olfactory, and quality of life improvements compared with nonsmokers [4–7, 17, 18]. Moreover, endoscopic abnormalities and significant increased revision ESS rates were frequently encountered in smokers compared to nonsmokers [5, 6, 19]. Outcomes may be associated with volume of smoking: a recent prospective large study confirmed that high-volume smokers displayed statistical significant poorer postoperative endoscopic scores compared to low-volume smokers or nonsmokers [20]. The relationship between SHS and outcome has been intensively studied in children [21]. Following ESS in children with SHS exposure ciliary regeneration was significantly impaired and symptomatic improvement drastically reduced [21, 22].

The most frequent symptoms after CS exposure are nasal congestion and rhinorrhea, explained through interference with ciliary beat frequency (CBF) and mucociliary clearance (MCC) [1, 2, 23]. Studies have provided conflicting results: Zhou et al. [23] demonstrated that cigarette smoke condensated (CSM) yielded increased CBF and MCC, while Cohen et al. [24] found that CSM determined decreased CBF. However, Zhou et al. [23] used low concentrations of CSM while Cohen et al. [24] used high CSM concentrations. Thus lower CSM concentrations increase CBF and MCC; high CSM concentrations inhibit both CBF and MCC [25].

Previous studies demonstrated the immunosuppressive effect of CS on nasal sinus epithelium [25]. Won et al. [26] found that CS decreases interleukin-8 (IL-8) and human *β*-defensin (HBD-2) in sinus cell cultures. This study suggests that CS might act as an immunosuppressive agent on nasal sinus innate immunity.

Balaji [27] in study on healing of oral mucosa exposed to CS suggested that the underlying healing pathomechanism of a wound exposed to CS is unknown. Nevertheless, the effect of nicotine through catecholamine release in increasing of platelet adhesiveness, microvascular occlusion, and tissue hypoxia has been demonstrated [28].

In this study we demonstrated that cigarette smoke induces sinonasal mucosa wound changes and delays in healing. After CS exposure the animals with sinonasal injuries present microscopic lesions of the nasal mucosa epithelium, of the glandular tissue, and of the associated lamina propria. Within the surface epithelium we demonstrated progressive injuries represented initially by loss of cilia and goblet cell hyperplasia, followed from day 14 by hyperplasic/dysplastic epithelium. Hyperplasic lesions were represented by multifocal micropapillary structures supported by a discrete connective tissue stroma. Normal evolution of wound healing consists of squamous epithelium metaplasia, moderate fibrosis in the lamina propria, which has a diffuse character in the nasal mucosa.

The changes found in lamina propria are represented by fibroplastic lesions starting from day 14 associated with hypertrophy of the serous glands starting on day 5. Besides those features, we found profuse leukocyte infiltration, initially represented by neutrophils and macrophages and subsequently by eosinophils.

Animals exposed at CS for 42 days presented marked fibroplasia in the lamina propria, which is associated with airway stenosis or serous glands obstruction.

The pathophysiology of immunologic alterations occasioned by CS exposure remains poorly understood. In animal experiments CS exposure yielded enhanced neutrophilic airway inflammation characterized by high IL-17 levels [29]. In an experimental asthma model it was found that CS could induce additive effects on bronchial inflammation and thus enable airway remodeling [30]. CS exposure raises goblet cell number and size with resultant increased secretion [29, 30].

Several experiments demonstrated that CS exposure and ovalbumin (OVA) sensitization produced raised expression of Th1 and Th2 cytokines with increased eosinophilic infiltration or else airway remodeling [30, 31]. One study found that long-term CS exposure induced enhanced eosinophilic infiltration and upregulation of inflammatory cytokines, while short-term CS exposure yielded decreased inflammatory changes [32]. Numerous studies have found that CS exposure exacerbates allergic inflammation through a dendritic cell- (DC-) associated pathway [32, 33]. In a murine model of nasal polyposis, Lee et al. [34] found that long-term CS exposure produced additive effects on eosinophil and mast cell infiltration and airway remodeling in the mouse nasal mucosa. In their experiment long-term CS exposure increased goblet cell number and thickness of subepithelial fibrosis. These authors demonstrated that CS exposure produced overexpression of Th1, Th2, and Th17 cytokines [34]. Several studies found that CS exposure upregulates VEGF levels in the lower animal and human airways, presenting a good association with HIF-1*α* [35, 36]. HIF-1*α* is able to facilitate the hypoxia-induced conversion of epithelial cells to mesenchymal cells [35, 36]. Lee et al. [34] demonstrated that CS exposure intensely enhanced VEGF and HIF-1*α* overexpression.

## 5. Conclusion

In this experimental model of nasal wound healing we demonstrated the deleterious effects of chronic CS exposure. The adverse effects of CS exposure in rats with nasal wound are firstly a postponement of the healing process and secondly the persistence of inflammation which becomes chronic.
